# 1-Dichloro­acetyl-*r*-2,*c*-6-bis­(4-methoxy­phen­yl)-*t*-3-methyl­piperidin-4-one

**DOI:** 10.1107/S1600536809054713

**Published:** 2010-01-09

**Authors:** K. Ravichandran, P. Ramesh, P. Sakthivel, S. Ponnuswamy, M. N. Ponnuswamy

**Affiliations:** a Centre of Advanced Study in Crystallography and Biophysics, University of Madras, Guindy Campus, Chennai 600 025, India; b Department of Chemistry, Government Arts College (Autonomous), Coimbatore 641 018, India

## Abstract

In the title compound, C_22_H_23_Cl_2_NO_4_, the piperidine ring adopts a distorted boat conformation. The meth­oxy groups lie in the plane of the benzene rings to which they are attached. The benzene rings are oriented at angles of 84.3 (1) and 76.8 (1)° with respect to the best plane through the piperidine ring. The crystal packing is stabilized by intermolecular C—H⋯O inter­actions.

## Related literature

For general background to piperidine derivatives, see: Perumal *et al.* (2001 ▶); Dimmock *et al.* (2001 ▶). For asymmetry parameters, see: Nardelli (1983 ▶). For puckering parameters, see: Cremer & Pople (1975 ▶). For hydrogen-bond motifs, see: Bernstein *et al.* (1995 ▶).
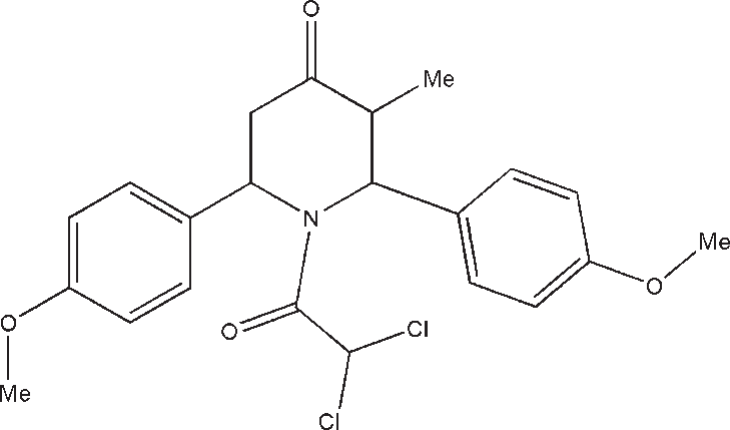


## Experimental

### 

#### Crystal data

C_22_H_23_Cl_2_NO_4_
*M* *_r_* = 436.31
Monoclinic, 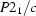

*a* = 19.3021 (16) Å
*b* = 10.5886 (9) Å
*c* = 10.3241 (10) Å
β = 91.445 (5)°
*V* = 2109.4 (3) Å^3^
*Z* = 4
Mo *K*α radiation
μ = 0.34 mm^−1^
*T* = 293 K
0.25 × 0.24 × 0.23 mm

#### Data collection

Bruker SMART APEXII area-detector diffractometer
Absorption correction: multi-scan (*SADABS*; Bruker, 2008 ▶) *T*_min_ = 0.919, *T*_max_ = 0.926
17272 measured reflections
5140 independent reflections
2632 reflections with *I* > 2σ(*I*)
*R*_int_ = 0.050

#### Refinement

*R*[*F* ^2^ > 2σ(*F* ^2^)] = 0.055
*wR*(*F* ^2^) = 0.210
*S* = 1.05
5140 reflections
265 parameters
H-atom parameters constrained
Δρ_max_ = 0.37 e Å^−3^
Δρ_min_ = −0.34 e Å^−3^

### 

Data collection: *APEX2* (Bruker, 2008 ▶); cell refinement: *SAINT* (Bruker, 2008 ▶); data reduction: *SAINT*; program(s) used to solve structure: *SHELXS97* (Sheldrick, 2008 ▶); program(s) used to refine structure: *SHELXL97* (Sheldrick, 2008 ▶); molecular graphics: *ORTEP-3* (Farrugia, 1997 ▶); software used to prepare material for publication: *SHELXL97* and *PLATON* (Spek, 2009 ▶).

## Supplementary Material

Crystal structure: contains datablocks global, I. DOI: 10.1107/S1600536809054713/bt5145sup1.cif

Structure factors: contains datablocks I. DOI: 10.1107/S1600536809054713/bt5145Isup2.hkl

Additional supplementary materials:  crystallographic information; 3D view; checkCIF report

## Figures and Tables

**Table 1:** Hydrogen-bond geometry (Å, °)

*D*—H⋯*A*	*D*—H	H⋯*A*	*D*⋯*A*	*D*—H⋯*A*
C6—H6⋯O1^i^	0.98	2.31	3.264 (4)	164
C8—H8⋯O1^i^	0.98	2.52	3.363 (4)	144
C18—H18⋯O1^i^	0.93	2.57	3.391 (4)	147

Symmetry code: (i) 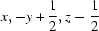.

**Fig. 1.**
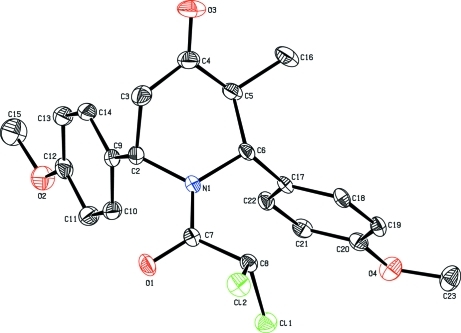
Perspective view of the molecule showing the thermal ellipsoids are drawn at 30% probability level. H atoms have been omitted for clarity.

**Fig. 2.**
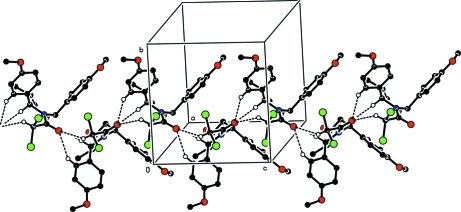
The crystal packing of the molecules viewed down a–axis. H atoms not involved in hydrogen bonding have been omitted for clarity.

## supplementary crystallographic information

### Comment

Piperidine derivatives are the valued heterocyclic compounds in the field of medicinal chemistry. piperidin-4-ones are reported to possess analgesic, anti-inflammatory, central nervous system (CNS), local anaesthetic, anticancer and antimicrobial activity (Perumal *et al.* (2001); Dimmock *et al.*,  2001). The crystallographic study of the title compound has been carried out to establish the molecular structure

The *ORTEP* diagram of the title compound is shown in Fig.1. The piperidine ring in the molecule adopts a distorted boat conformation with the puckering parameters (Cremer & Pople, 1975) and the asymmetry parameters (Nardelli, 1983) are: q_2_ = 0.685 (3) Å, q_3_ = 0.039 (3) Å, φ_2_ = 72.1 (3)° and Δ_s_(C2 & C5)= 14.9 (3)°. The methoxy groups lie in the plane of phenyl rings and these phenyl rings are oriented at angles of 84.3 (1)° and 76.8 (1)° with best plane of piperidine ring.The sum of the bond angles around the atom N1(359.7°) of the piperidine ring in the molecule is in accordance with *sp*^2^ hybridization.

The crystal packing is stabilized by C—H···O types of intra and intermolecular interactions, which link the molecules into a chain extending along the c axis. Atoms C6, C18 and C4 of the molecule at (x, y, z) donate a proton to trifurcated acceptor atom O1 of the molecule at (x, 1/2 - y, -1/2 + z). Intermolecular interactions C6—H6···O1 and C18—H18···O1 form C5 & C7 zigzag chains (Bernstein *et al.*, 1995), whereas the other interaction C8—H8···O1 forms a C4 one-dimensional chain, running along the c axis, as shown in Fig. 2.

### Experimental

To a solution of r-2,c-6-bis(4-methoxyphenyl)-t-3-methylpiperidin-4-one (1.625 g) in anhydrous benzene (60 ml) was added triethylamine (2.1 ml) and dichloroacetylchloride (1.42 ml). The reaction mixture was allowed to stirr at room teperature for 2hrs. The resulting solution was washed with sodium bicarbonate solution (10%) and water. The organic layer was dried over anhydrous sodium sulfate, evaporated and crystallized from benzene: pet-ether (60–80°C) in the ratio of 9:1.

### Refinement

H atoms were positioned geometrically (C—H=0.93–0.98 Å) and allowed to ride on their parent atoms, with 1.5*U*_eq_(C) for methyl H and 1.2 *U*_eq_(C) for other H atoms.

### Crystal data

**Table:**

C_22_H_23_Cl_2_NO_4_	*F*(000) = 912
*M**_r_* = 436.31	*D*_x_ = 1.374 Mg m^−^^3^
Monoclinic, *P*2_1_/*c*	Mo *K*α radiation, λ = 0.71073 Å
Hall symbol: -P 2ybc	Cell parameters from 2564 reflections
*a* = 19.3021 (16) Å	θ = 1.1–28.7°
*b* = 10.5886 (9) Å	µ = 0.34 mm^−^^1^
*c* = 10.3241 (10) Å	*T* = 293 K
β = 91.445 (5)°	Block, colorless
*V* = 2109.4 (3) Å^3^	0.25 × 0.24 × 0.23 mm
*Z* = 4	

### Data collection

**Table:**

Bruker SMART APEXII area-detector diffractometer	5140 independent reflections
Radiation source: fine-focus sealed tube	2632 reflections with *I* > 2σ(*I*)
graphite	*R*_int_ = 0.050
ω and φ scans	θ_max_ = 28.7°, θ_min_ = 1.1°
Absorption correction: multi-scan (*SADABS*; Bruker, 2008)	*h* = −25→25
*T*_min_ = 0.919, *T*_max_ = 0.926	*k* = −13→14
17272 measured reflections	*l* = −13→10

### Refinement

**Table:**

Refinement on *F*^2^	Primary atom site location: structure-invariant direct methods
Least-squares matrix: full	Secondary atom site location: difference Fourier map
*R*[*F*^2^ > 2σ(*F*^2^)] = 0.055	Hydrogen site location: inferred from neighbouring sites
*wR*(*F*^2^) = 0.210	H-atom parameters constrained
*S* = 1.05	*w* = 1/[σ^2^(*F*_o_^2^) + (0.1035*P*)^2^ + 0.3613*P*] where *P* = (*F*_o_^2^ + 2*F*_c_^2^)/3
5140 reflections	(Δ/σ)_max_ = 0.001
265 parameters	Δρ_max_ = 0.37 e Å^−^^3^
0 restraints	Δρ_min_ = −0.34 e Å^−^^3^

### Special details

**Table:**

Geometry. All e.s.d.'s (except the e.s.d. in the dihedral angle between two l.s. planes) are estimated using the full covariance matrix. The cell e.s.d.'s are taken into account individually in the estimation of e.s.d.'s in distances, angles and torsion angles; correlations between e.s.d.'s in cell parameters are only used when they are defined by crystal symmetry. An approximate (isotropic) treatment of cell e.s.d.'s is used for estimating e.s.d.'s involving l.s. planes.
Refinement. Refinement of *F*^2^ against ALL reflections. The weighted *R*-factor *wR* and goodness of fit *S* are based on *F*^2^, conventional *R*-factors *R* are based on *F*, with *F* set to zero for negative *F*^2^. The threshold expression of *F*^2^ > σ(*F*^2^) is used only for calculating *R*-factors(gt) *etc*. and is not relevant to the choice of reflections for refinement. *R*-factors based on *F*^2^ are statistically about twice as large as those based on *F*, and *R*- factors based on ALL data will be even larger.

### Fractional atomic coordinates and isotropic or equivalent isotropic displacement parameters (Å^2^)

**Table:**

	*x*	*y*	*z*	*U*_iso_*/*U*_eq_	
Cl1	0.06236 (4)	0.37763 (8)	0.02208 (10)	0.0600 (3)	
Cl2	0.10261 (5)	0.12850 (8)	−0.06850 (10)	0.0618 (3)	
O1	0.18680 (12)	0.2296 (2)	0.1496 (2)	0.0611 (7)	
O2	0.37098 (13)	0.6870 (3)	0.4740 (3)	0.0714 (8)	
O3	0.43242 (15)	0.3729 (4)	−0.1711 (4)	0.1125 (13)	
O4	0.06912 (12)	0.8442 (2)	−0.1071 (3)	0.0607 (7)	
N1	0.24905 (12)	0.3470 (2)	0.0117 (2)	0.0378 (6)	
C2	0.31270 (15)	0.3183 (3)	0.0888 (3)	0.0433 (8)	
H2	0.3025	0.2422	0.1388	0.052*	
C3	0.36826 (17)	0.2806 (3)	−0.0059 (4)	0.0568 (9)	
H3A	0.4110	0.2633	0.0423	0.068*	
H3B	0.3541	0.2032	−0.0493	0.068*	
C4	0.38194 (18)	0.3784 (4)	−0.1054 (4)	0.0617 (10)	
C5	0.32820 (16)	0.4799 (3)	−0.1204 (3)	0.0481 (8)	
H5	0.3364	0.5402	−0.0496	0.058*	
C6	0.25383 (14)	0.4284 (3)	−0.1064 (3)	0.0379 (7)	
H6	0.2431	0.3755	−0.1821	0.046*	
C7	0.19106 (15)	0.2871 (3)	0.0469 (3)	0.0407 (7)	
C8	0.12870 (15)	0.2872 (3)	−0.0465 (3)	0.0413 (7)	
H8	0.1416	0.3238	−0.1297	0.050*	
C9	0.33116 (14)	0.4193 (3)	0.1869 (3)	0.0424 (7)	
C10	0.28014 (16)	0.4676 (3)	0.2662 (3)	0.0502 (8)	
H10	0.2348	0.4393	0.2548	0.060*	
C11	0.29471 (17)	0.5556 (4)	0.3605 (3)	0.0562 (9)	
H11	0.2595	0.5863	0.4117	0.067*	
C12	0.36200 (18)	0.5987 (3)	0.3792 (3)	0.0530 (9)	
C13	0.41367 (17)	0.5519 (4)	0.3030 (4)	0.0584 (10)	
H13	0.4591	0.5795	0.3151	0.070*	
C14	0.39748 (16)	0.4635 (4)	0.2082 (3)	0.0543 (9)	
H14	0.4327	0.4328	0.1571	0.065*	
C15	0.4393 (2)	0.7283 (4)	0.5030 (5)	0.0878 (14)	
H15A	0.4678	0.6569	0.5255	0.132*	
H15B	0.4389	0.7863	0.5744	0.132*	
H15C	0.4577	0.7698	0.4286	0.132*	
C16	0.3347 (2)	0.5526 (4)	−0.2460 (4)	0.0691 (11)	
H16A	0.3821	0.5770	−0.2566	0.104*	
H16B	0.3061	0.6267	−0.2436	0.104*	
H16C	0.3199	0.5001	−0.3173	0.104*	
C17	0.20227 (14)	0.5359 (3)	−0.1064 (3)	0.0348 (7)	
C18	0.15426 (15)	0.5514 (3)	−0.2067 (3)	0.0399 (7)	
H18	0.1529	0.4928	−0.2738	0.048*	
C19	0.10828 (16)	0.6511 (3)	−0.2103 (3)	0.0428 (7)	
H19	0.0759	0.6581	−0.2781	0.051*	
C20	0.11057 (16)	0.7406 (3)	−0.1126 (3)	0.0425 (7)	
C21	0.15861 (16)	0.7270 (3)	−0.0128 (3)	0.0456 (8)	
H21	0.1608	0.7871	0.0528	0.055*	
C22	0.20341 (16)	0.6263 (3)	−0.0083 (3)	0.0419 (7)	
H22	0.2348	0.6183	0.0610	0.050*	
C23	0.0234 (2)	0.8678 (3)	−0.2145 (5)	0.0703 (11)	
H23A	0.0497	0.8761	−0.2917	0.105*	
H23B	−0.0018	0.9444	−0.1999	0.105*	
H23C	−0.0085	0.7987	−0.2243	0.105*	

### Atomic displacement parameters (Å^2^)

**Table:**

	*U*^11^	*U*^22^	*U*^33^	*U*^12^	*U*^13^	*U*^23^
Cl1	0.0590 (5)	0.0573 (5)	0.0641 (7)	0.0081 (4)	0.0103 (4)	0.0020 (4)
Cl2	0.0749 (6)	0.0441 (5)	0.0665 (7)	−0.0149 (4)	0.0003 (5)	−0.0053 (4)
O1	0.0674 (15)	0.0703 (16)	0.0452 (15)	−0.0148 (12)	−0.0051 (12)	0.0270 (13)
O2	0.0731 (17)	0.0837 (18)	0.0570 (18)	−0.0107 (14)	−0.0054 (14)	−0.0164 (15)
O3	0.0646 (18)	0.161 (3)	0.114 (3)	0.0281 (19)	0.0481 (19)	0.042 (2)
O4	0.0657 (15)	0.0398 (12)	0.076 (2)	0.0079 (11)	−0.0022 (14)	−0.0061 (12)
N1	0.0424 (13)	0.0422 (14)	0.0288 (15)	0.0000 (11)	0.0011 (11)	0.0049 (11)
C2	0.0434 (17)	0.0500 (18)	0.037 (2)	0.0071 (14)	−0.0012 (14)	0.0052 (15)
C3	0.0512 (19)	0.068 (2)	0.051 (2)	0.0129 (17)	0.0018 (16)	−0.0085 (19)
C4	0.0458 (19)	0.088 (3)	0.052 (2)	−0.0024 (18)	0.0091 (17)	−0.006 (2)
C5	0.0497 (18)	0.058 (2)	0.037 (2)	−0.0090 (15)	0.0100 (14)	−0.0009 (16)
C6	0.0457 (16)	0.0459 (17)	0.0223 (16)	−0.0058 (13)	0.0028 (12)	−0.0004 (13)
C7	0.0467 (17)	0.0386 (16)	0.0367 (19)	−0.0007 (13)	0.0002 (14)	0.0030 (14)
C8	0.0463 (16)	0.0394 (15)	0.0382 (19)	−0.0059 (13)	0.0011 (14)	0.0018 (13)
C9	0.0397 (16)	0.0568 (19)	0.0307 (18)	0.0009 (14)	−0.0018 (13)	0.0029 (15)
C10	0.0420 (17)	0.068 (2)	0.041 (2)	−0.0006 (15)	−0.0010 (14)	−0.0059 (17)
C11	0.0478 (19)	0.077 (2)	0.043 (2)	0.0032 (17)	0.0017 (15)	−0.0102 (19)
C12	0.060 (2)	0.065 (2)	0.033 (2)	−0.0035 (17)	−0.0043 (16)	−0.0014 (17)
C13	0.0459 (19)	0.083 (3)	0.046 (2)	−0.0115 (18)	−0.0030 (16)	0.001 (2)
C14	0.0411 (17)	0.083 (3)	0.038 (2)	−0.0005 (17)	0.0037 (14)	−0.0004 (19)
C15	0.086 (3)	0.098 (3)	0.078 (3)	−0.025 (3)	−0.013 (3)	−0.022 (3)
C16	0.079 (3)	0.078 (3)	0.051 (3)	−0.019 (2)	0.019 (2)	0.010 (2)
C17	0.0474 (16)	0.0344 (15)	0.0228 (16)	−0.0052 (12)	0.0054 (12)	0.0021 (12)
C18	0.0579 (18)	0.0344 (15)	0.0276 (18)	−0.0022 (13)	0.0029 (14)	−0.0015 (13)
C19	0.0543 (18)	0.0383 (16)	0.0356 (19)	−0.0048 (13)	−0.0026 (14)	0.0099 (14)
C20	0.0512 (17)	0.0336 (16)	0.043 (2)	−0.0036 (13)	0.0078 (15)	0.0030 (14)
C21	0.0567 (18)	0.0443 (17)	0.036 (2)	−0.0033 (15)	0.0074 (15)	−0.0106 (15)
C22	0.0478 (17)	0.0485 (18)	0.0294 (18)	−0.0010 (14)	−0.0010 (13)	−0.0004 (14)
C23	0.068 (2)	0.051 (2)	0.090 (3)	0.0093 (18)	−0.012 (2)	0.014 (2)

### Geometric parameters (Å, °)

**Table:**

Cl1—C8	1.762 (3)	C10—C11	1.372 (5)
Cl2—C8	1.768 (3)	C10—H10	0.9300
O1—C7	1.228 (4)	C11—C12	1.385 (4)
O2—C12	1.362 (4)	C11—H11	0.9300
O2—C15	1.415 (4)	C12—C13	1.378 (5)
O3—C4	1.203 (4)	C13—C14	1.383 (5)
O4—C20	1.360 (4)	C13—H13	0.9300
O4—C23	1.422 (4)	C14—H14	0.9300
N1—C7	1.344 (4)	C15—H15A	0.9600
N1—C2	1.479 (4)	C15—H15B	0.9600
N1—C6	1.498 (4)	C15—H15C	0.9600
C2—C9	1.509 (4)	C16—H16A	0.9600
C2—C3	1.523 (4)	C16—H16B	0.9600
C2—H2	0.9800	C16—H16C	0.9600
C3—C4	1.487 (5)	C17—C18	1.382 (4)
C3—H3A	0.9700	C17—C22	1.394 (4)
C3—H3B	0.9700	C18—C19	1.379 (4)
C4—C5	1.499 (5)	C18—H18	0.9300
C5—C16	1.516 (5)	C19—C20	1.384 (4)
C5—C6	1.546 (4)	C19—H19	0.9300
C5—H5	0.9800	C20—C21	1.377 (4)
C6—C17	1.512 (4)	C21—C22	1.372 (4)
C6—H6	0.9800	C21—H21	0.9300
C7—C8	1.523 (4)	C22—H22	0.9300
C8—H8	0.9800	C23—H23A	0.9600
C9—C14	1.375 (4)	C23—H23B	0.9600
C9—C10	1.393 (4)	C23—H23C	0.9600
			
C12—O2—C15	117.7 (3)	C10—C11—H11	120.1
C20—O4—C23	117.6 (3)	C12—C11—H11	120.1
C7—N1—C2	116.4 (2)	O2—C12—C13	125.2 (3)
C7—N1—C6	124.0 (2)	O2—C12—C11	115.3 (3)
C2—N1—C6	119.3 (2)	C13—C12—C11	119.4 (3)
N1—C2—C9	113.3 (2)	C12—C13—C14	119.5 (3)
N1—C2—C3	107.3 (3)	C12—C13—H13	120.2
C9—C2—C3	117.3 (3)	C14—C13—H13	120.2
N1—C2—H2	106.1	C9—C14—C13	122.4 (3)
C9—C2—H2	106.1	C9—C14—H14	118.8
C3—C2—H2	106.1	C13—C14—H14	118.8
C4—C3—C2	113.6 (3)	O2—C15—H15A	109.5
C4—C3—H3A	108.8	O2—C15—H15B	109.5
C2—C3—H3A	108.8	H15A—C15—H15B	109.5
C4—C3—H3B	108.8	O2—C15—H15C	109.5
C2—C3—H3B	108.8	H15A—C15—H15C	109.5
H3A—C3—H3B	107.7	H15B—C15—H15C	109.5
O3—C4—C3	121.1 (4)	C5—C16—H16A	109.5
O3—C4—C5	123.0 (4)	C5—C16—H16B	109.5
C3—C4—C5	115.9 (3)	H16A—C16—H16B	109.5
C4—C5—C16	112.4 (3)	C5—C16—H16C	109.5
C4—C5—C6	112.3 (3)	H16A—C16—H16C	109.5
C16—C5—C6	110.9 (3)	H16B—C16—H16C	109.5
C4—C5—H5	107.0	C18—C17—C22	117.5 (3)
C16—C5—H5	107.0	C18—C17—C6	121.2 (3)
C6—C5—H5	107.0	C22—C17—C6	121.3 (3)
N1—C6—C17	112.3 (2)	C19—C18—C17	122.0 (3)
N1—C6—C5	110.8 (2)	C19—C18—H18	119.0
C17—C6—C5	110.3 (2)	C17—C18—H18	119.0
N1—C6—H6	107.7	C18—C19—C20	119.7 (3)
C17—C6—H6	107.7	C18—C19—H19	120.1
C5—C6—H6	107.7	C20—C19—H19	120.1
O1—C7—N1	122.8 (3)	O4—C20—C21	116.0 (3)
O1—C7—C8	118.6 (3)	O4—C20—C19	125.1 (3)
N1—C7—C8	118.6 (3)	C21—C20—C19	118.9 (3)
C7—C8—Cl1	108.5 (2)	C22—C21—C20	121.3 (3)
C7—C8—Cl2	107.4 (2)	C22—C21—H21	119.4
Cl1—C8—Cl2	111.14 (16)	C20—C21—H21	119.4
C7—C8—H8	109.9	C21—C22—C17	120.7 (3)
Cl1—C8—H8	109.9	C21—C22—H22	119.7
Cl2—C8—H8	109.9	C17—C22—H22	119.7
C14—C9—C10	116.7 (3)	O4—C23—H23A	109.5
C14—C9—C2	123.5 (3)	O4—C23—H23B	109.5
C10—C9—C2	119.7 (3)	H23A—C23—H23B	109.5
C11—C10—C9	122.1 (3)	O4—C23—H23C	109.5
C11—C10—H10	119.0	H23A—C23—H23C	109.5
C9—C10—H10	119.0	H23B—C23—H23C	109.5
C10—C11—C12	119.8 (3)		
			
C7—N1—C2—C9	−101.3 (3)	N1—C2—C9—C10	46.9 (4)
C6—N1—C2—C9	84.8 (3)	C3—C2—C9—C10	172.8 (3)
C7—N1—C2—C3	127.6 (3)	C14—C9—C10—C11	0.5 (5)
C6—N1—C2—C3	−46.3 (3)	C2—C9—C10—C11	177.3 (3)
N1—C2—C3—C4	57.2 (4)	C9—C10—C11—C12	−0.2 (6)
C9—C2—C3—C4	−71.6 (4)	C15—O2—C12—C13	−4.4 (6)
C2—C3—C4—O3	166.1 (4)	C15—O2—C12—C11	176.0 (3)
C2—C3—C4—C5	−15.7 (4)	C10—C11—C12—O2	179.3 (3)
O3—C4—C5—C16	14.5 (5)	C10—C11—C12—C13	−0.3 (6)
C3—C4—C5—C16	−163.6 (3)	O2—C12—C13—C14	−179.0 (3)
O3—C4—C5—C6	140.2 (4)	C11—C12—C13—C14	0.6 (6)
C3—C4—C5—C6	−37.8 (4)	C10—C9—C14—C13	−0.2 (5)
C7—N1—C6—C17	57.7 (4)	C2—C9—C14—C13	−176.9 (3)
C2—N1—C6—C17	−128.9 (3)	C12—C13—C14—C9	−0.3 (6)
C7—N1—C6—C5	−178.4 (3)	N1—C6—C17—C18	−122.7 (3)
C2—N1—C6—C5	−5.1 (4)	C5—C6—C17—C18	113.1 (3)
C4—C5—C6—N1	48.3 (4)	N1—C6—C17—C22	60.2 (3)
C16—C5—C6—N1	174.9 (3)	C5—C6—C17—C22	−64.0 (3)
C4—C5—C6—C17	173.3 (3)	C22—C17—C18—C19	−0.8 (4)
C16—C5—C6—C17	−60.1 (3)	C6—C17—C18—C19	−178.0 (3)
C2—N1—C7—O1	12.7 (4)	C17—C18—C19—C20	1.4 (4)
C6—N1—C7—O1	−173.7 (3)	C23—O4—C20—C21	174.2 (3)
C2—N1—C7—C8	−165.4 (3)	C23—O4—C20—C19	−5.0 (4)
C6—N1—C7—C8	8.2 (4)	C18—C19—C20—O4	178.5 (3)
O1—C7—C8—Cl1	69.3 (3)	C18—C19—C20—C21	−0.7 (4)
N1—C7—C8—Cl1	−112.5 (3)	O4—C20—C21—C22	−179.8 (3)
O1—C7—C8—Cl2	−50.9 (3)	C19—C20—C21—C22	−0.6 (4)
N1—C7—C8—Cl2	127.3 (3)	C20—C21—C22—C17	1.2 (4)
N1—C2—C9—C14	−136.5 (3)	C18—C17—C22—C21	−0.5 (4)
C3—C2—C9—C14	−10.6 (5)	C6—C17—C22—C21	176.7 (3)

### Hydrogen-bond geometry (Å, °)

**Table:**

*D*—H···*A*	*D*—H	H···*A*	*D*···*A*	*D*—H···*A*
C6—H6···O1^i^	0.98	2.31	3.264 (4)	164
C8—H8···O1^i^	0.98	2.52	3.363 (4)	144
C18—H18···O1^i^	0.93	2.57	3.391 (4)	147

Symmetry codes: (i) *x*, −*y*+1/2, *z*−1/2.
